# Estrategias para el desarrollo de habilidades en práctica basada en la evidencia a través del currículo de enfermería*

**DOI:** 10.15649/cuidarte.3089

**Published:** 2023-12-24

**Authors:** Skarlet Marcell Vásquez, Adriana Patricia Bonilla-Marciales, Mary Luz Jaimes-Valencia

**Affiliations:** 1 . Universidad Autónoma de Bucaramanga. Bucaramanga, Colombia. E-mail: svasquez196@unab.edu.co Universidad Autónoma de Bucaramanga Universidad Autónoma de Bucaramanga Bucaramanga Colombia svasquez196@unab.edu.co; 2 . Universidad Autónoma de Bucaramanga. Bucaramanga, Colombia. E-mail: abonilla712@unab.edu.co Universidad Autónoma de Bucaramanga Universidad Autónoma de Bucaramanga Bucaramanga Colombia abonilla712@unab.edu.co; 3 . Universidad Autónoma de Bucaramanga. Bucaramanga, Colombia. E-mail: mjaimes239@unab.edu.co Universidad Autónoma de Bucaramanga Universidad Autónoma de Bucaramanga Bucaramanga Colombia mjaimes239@unab.edu.co

**Keywords:** Curriculum, Educación Basada en Competencias, Estudiantes de Enfermería, Práctica Clínica Basada en la Evidencia, Curriculum, Competency-Based Education, Students Nursing, Evidence-Based Practice, Currículo, Educagáo Baseada em Competencias, Estudantes de Enfermagem, Prática Clínica Baseada em Evidencias

## Abstract

**Introducción:**

Mundialmente, se reconoce la enseñanza de la práctica basada en evidencia como una estrategia que contribuye a mejorar la calidad de la atención en salud.

**Objetivo:**

Identificar el nivel de competencias para la práctica basada en evidencia en estudiantes de enfermería de acuerdo con las características del currículo cursado en una universidad colombiana.

**Materiales y Métodos:**

Estudio de corte transversal, con mediciones a estudiantes de enfermería en tres periodos (N=125): currículo-1 sin formación en investigación (n=24); currículo-2 con formación en investigación (n=55); currículo-3 con formación en práctica basada en evidencia (n=46). El desenlace principal fue competencias para la práctica basada en evidencia medido por actitudes, conocimientos y habilidades.

**Resultados:**

El puntaje global de competencias fue 4,08 (Rango intercuartílico=3,84- 4,32), no se observaron diferencias estadísticamente significativas entre los puntajes obtenidos por estudiantes de los tres currículos, sin embargo, las competencias de conocimientos para la práctica basada en evidencia fueron significativamente mayores en el grupo del currículo 3.

**Discusión:**

Algunos autores han descrito la relación entre el tiempo de las transformaciones curriculares y el desarrollo de competencias progresivas, como un proceso escalonado cuyos resultados se reflejan al menos un ciclo curricular completo después de su implementación.

**Conclusiones:**

Los ajustes curriculares realizados en esta institución han permitido mejorar la percepción de los conocimientos para la práctica basada en evidencia en estudiantes de enfermería, sin embargo, es necesario seguir trabajando en estrategias pedagógicas eficaces que mejoren las habilidades para su implementación. Esta información provee orientación para planeación curricular.

## Abstract (en)

**Introduction:**

Teaching evidence-based practice is a recognized worldwide strategy for improving health care quality. Objective: To identify the level of evidence-based practice competencies of nursing students according to the characteristics of the curriculum followed in a Colombian university.

**Materials and Methods:**

Cross-sectional study measuring nursing students in three periods (N=125): Curriculum-1 without research training (n=24); Curriculum-2 with research training (n=55); Curriculum-3 with evidence-based practice training (n=46). The primary outcome was evidence-based practice competencies measured by attitude, knowledge, and skills.

**Results:**

The global competence score was 4.08 (interquartile range = 3.84-4.32), and no statistically significant differences were observed between the scores obtained by students in the three curricula. However, knowledge competencies for evidence-based practice were significantly higher in the Curriculum 3 group.

**Discussion:**

Some authors have described the relationship between the timing of curricular transformations and the development of progressive competencies as a staggered process whose results are reflected in at least one complete curricular cycle after its implementation.

**Conclusions:**

The curricular adjustments made at this institution have improved the perception of knowledge in evidence-based practice among nursing students; however, it is necessary to continue working on effective pedagogical strategies that enhance the skills for its implementation. This information provides guidance for curriculum planning.

## Resumo

**Introdugáo:**

Mundialmente, o ensino da prática baseada em evidencias é reconhecido como uma estratégia que contribui para a melhoria da qualidade dos cuidados de saúde.

**Objetivo:**

Identificar o nível de competencias para a prática baseada em evidencias em estudantes de enfermagem segundo as características do currículo estudado em uma universidade colombiana.

**Materiais e Métodos:**

Estudo transversal, com mensurales de estudantes de enfermagem em tres períodos (N=125): currículo-1 sem formagáo em pesquisa (n=24); currículo-2 com formagáo em pesquisa (n=55); currículo-3 com formagáo em prática baseada em evidencias (n=46). O resultado primário foram competencias para a prática baseada em evidencias medidas por atitudes, conhecimentos e habilidades.

**Resultados:**

A pontuagáo geral de competencia foi de 4,08 (intervalo interquartil=3,84-4,32), náo foram observadas diferengas estatisticamente significativas entre as pontuagóes obtidas pelos alunos dos tres currículos, porém, as competencias de conhecimento para a prática baseada em evidencias foram significativamente maiores no grupo curricular 3.

**Discussao:**

Alguns autores descreveram a relagáo entre o momento das transformagóes curriculares e o desenvolvimento de competencias progressivas, como um processo gradual cujos resultados se refletem pelo menos um ciclo curricular completo após a sua implementagáo.

**Conclusóes:**

As adequagóes curriculares realizadas nesta instituigáo melhoraram a percepgáo do conhecimento para a prática baseada em evidencias nos estudantes de enfermagem, porém é necessário continuar trabalhando em estratégias pedagógicas eficazes que melhorem as competencias para sua implementagáo. Estas informagóes fornecem orientagáo para o planejamento curricular.

## Introducción

La enseñanza de la práctica basada en la evidencia (PBE) en los currículos de enfermería ha sido promovida mundialmente con el fin de garantizar la formación de profesionales capacitados para la toma de decisiones que contribuyan a mejorar la calidad de la atención en salud^1^^-^^3^.

A pesar de que educadores y estudiantes de enfermería reconocen su importancia, algunos estudios han reportado una baja implementación de la PBE, identificando barreras como: falta de conocimientos para utilizar la investigación en enfermería, falta de confianza en la enseñanza y falta de tiempo para incorporar los elementos necesarios para la enseñanza- aprendizaje de la PBE a nivel curricular y también para su posterior implementación en el ejercicio profesional^4^^-^^7^. Consecuentemente, los programas de formación de pregrado y posgrado de enfermería trabajan generando transformaciones curriculares continuas, que buscan incorporar estrategias pedagógicas para que los estudiantes alcancen de forma eficaz las competencias para la PBE^8^^-^^13^.

Actualmente en Colombia, dadas las disposiciones que regulan las políticas y acciones para la formación de profesionales de enfermería^14^, los currículos de la mayor parte de los programas certificados incluyen la preparación para la investigación en salud, sin embargo, los contenidos suelen limitarse al conocimiento de los métodos de investigación y bioestadística, y son insuficientes en desarrollar capacidades para el análisis crítico de la literatura y la toma de decisiones a partir de la misma^15^^,^^16^_._

Por ejemplo, en un estudio realizado en cuatro instituciones de educación superior en Santander, se observó que menos del 50% de los currículos de estas universidades incorporaban contenidos sobre el desarrollo de competencias para la toma de decisiones clínicas, elaboración de juicios clínicos, capacidades para implementar cambios y habilidades para la búsqueda de literatura científica. Adicionalmente, menos de un 15% de los educadores manifestaron desarrollar actividades de análisis crítico de la literatura relacionada con los casos de cuidado en las prácticas de enfermería^16^. Consistentemente, estudios realizados en universidades de otras regiones del país, han reportado un nivel medio-bajo en las actitudes, habilidades y prácticas, de las competencias para la PBE auto reportadas por los estudiantes de los programas de enfermería^17^^,^^18^.

A lo largo de 15 años de trayectoria del programa de enfermería de una institución en el Nororiente Colombiano que ofrece la formación de pregrado en ocho semestres académicos, se han tenido dos ajustes curriculares importantes; inicialmente para incorporar la formación en investigación en enfermería y más recientemente para incluir la formación en PBE. Sin embargo, el impacto de estos ajustes curriculares sobre las competencias para la PBE reportados por los estudiantes del programa no ha sido evaluado formalmente, por lo tanto, se propuso un estudio con el objetivo de identificar el nivel de competencias para la práctica basada en evidencia en estudiantes de enfermería de acuerdo con las características del currículo cursado en una universidad colombiana.

## Materiales y Métodos

Diseño: Se realizó un estudio de corte transversal, con mediciones a tres muestras de estudiantes de enfermería durante tres periodos curriculares.

Escenario: El estudio se realizó en una universidad privada ubicada en el Nororiente Colombiano, cuyo programa de enfermería tiene 15 años de antigüedad y tres periodos curriculares relevantes.

Para efectos del presente estudio, se denominó primer periodo curricular al currículo desarrollado en 2008 y vigente para nuevos ingresos hasta el segundo semestre de 2013 (Última cohorte con egresados hasta primer semestre de 2017). Este currículo tenía 164 créditos académicos (CA) (2128 horas de contenidos teóricos y 2160 horas de prácticas) distribuidos en 41 cursos, para desarrollar en 8 semestres. Desde el enfoque de formación en investigación, el plan de estudios incluía un curso de introducción a la salud pública y un curso de condicionantes en salud, sin embargo, no se tenían cursos de formación específica en investigación en enfermería (IE). Por lo que el periodo curricular se identificó como Currículo SIN formación IE.

De acuerdo con el plan de estudios, el curso de introducción a la salud pública hacia parte del primer semestre (Intensidad de 6 CA). En este curso se incluyeron contenidos relacionados con conceptos de salud-enfermedad, promoción y prevención, medidas de resumen e indicadores epidemiológicos. Por su parte, el curso de condicionantes en salud (Intensidad 6 CA) se ubicó en el segundo semestre, con el objetivo de exponer los contenidos relacionados con aspectos básicos del diseño y análisis de estudios epidemiológicos, principios de inferencia estadística y causal, perspectiva de determinantes sociales.

Se consideró como segundo periodo curricular, al periodo comprendido entre primer semestre del 2014 y segundo semestre de 2019. Durante este periodo se reforma el plan de estudios, pasando a 161 CA (2166 horas de contenidos teóricos y 2402 horas de prácticas) distribuidos en 38 cursos. En cuanto a la formación en IE, el cambio más importante estuvo representado en la adicción de 4 CA relacionados con los cursos de investigación en enfermería 1 e investigación en enfermería 2. Para efectos de este estudio, este segundo periodo se identificó como Currículo CON formación IE.

Los cursos de IE se ubicaron en los semestres sexto y séptimo, cada curso de 2 CA. Los contenidos en el curso de IE 1, incluían diseños de investigación cuantitativa y cualitativa, cálculo e interpretación de medidas de bioestadística, elaboración de un protocolo de IE. Para dar continuidad a estos contenidos, el curso de IE 2, abordaba uso de herramientas para la sistematización y el análisis de datos cuantitativos y cualitativos, estrategias para la validación de instrumentos de medición, así como la ejecución de un proyecto de IE.

Finalmente, se denominó tercer periodo curricular, al periodo comprendido entre el primer y segundo semestre de 2020. Aunque en este periodo no se ejecutó una reforma curricular en el sentido estricto, se modificaron los contenidos de estudio a nivel micro curricular. Consecuentemente, en términos de formación en investigación, se incluyó dentro de los cursos de IE, contenidos para el desarrollo de dos módulos de formación específica en PBE (estos módulos debían ser tomados de forma obligatoria). Estos módulos representan el 20% de la intensidad horaria para cada uno de los cursos de IE, e incluyen contenidos relacionados con epistemología de la PBE, etapas de la PBE, formulación de preguntas clínicas, búsqueda de información, lectura crítica, implementación y evaluación de la evidencia, desarrollados a través de estrategias pedagógicas como talleres, tutorías, cursos en modalidad híbrida (consulta asincrónica y permanente de contenidos). Este periodo se identificó como Currículo CON formación en PBE.

Población y muestra: Dada la naturaleza del estudio, se realizó un muestreo por conveniencia en el que se incluyeron todos los estudiantes que cumplieron con los siguientes criterios de selección: a) Tener matricula activa durante el semestre en que se realizó la medición, b) Estar cursando el último año del programa de enfermería (Séptimo u Octavo semestre), c) Dar consentimiento informado para su participación. Se excluyeron estudiantes que no respondieron la totalidad de preguntas del cuestionario. Para asegurar que los estudiantes seleccionados hubieran cursado exclusivamente uno de los tres currículos, las muestras fueron seleccionadas en fechas en las que la penúltima y última cohorte de estudiantes ingresada para el periodo curricular de interés estuviera cursando el séptimo u octavo semestre, respectivamente. Para el primer periodo curricular la muestra se recolectó en el primer semestre de 2017, en este grupo se incluyó exclusivamente a estudiantes que cursaban el octavo semestre debido a limitaciones para el acceso de las investigadoras a los estudiantes de séptimo semestre. Para el periodo curricular 2 la muestra se recolectó durante el segundo semestre de 2017, mientras que para el periodo curricular 3 la muestra se obtuvo en el segundo semestre de 2020.

Variables de interés: Las competencias sobre la PBE fueron el desenlace principal de este estudio. Estas competencias se midieron en tres dimensiones: 1) Actitudes, 2) Conocimientos y 3) Habilidades, con la escala CACH-PBE elaborada por María Ruzafa Martínez y colaboradores en el año 2015, validada para Colombia^18^^,^^19^. La escala cuenta con 13 ítems que evalúan la dimensión de actitudes, 6 ítems la dimensión de conocimientos y 6 ítems la dimensión de habilidades. Cada uno de estos ítems es autocalificado por el estudiante a través de una escala ordinal con valores entre 1 a 5, siendo 1 muy en desacuerdo y 5 muy de acuerdo. La puntuación obtenida en el total de los ítems se promedia para obtener el puntaje total de la escala, que se interpreta de forma numérica.

Aunque sus equivalentes cualitativos no han sido expresados por los autores, estudios previos interpretan puntajes inferiores a 3 como un nivel de competencias bajo, entre 3-4 nivel medio y superiores a 4 nivel alto^17^^,^^18^. Adicionalmente, el instrumento mide la autopercepción de 8 de ítems relacionados con la PBE (Como: actitud de los colegas, conocimientos en inglés, informática y bioestadística), para estos ítems el estudiante asigna un puntaje entre 0 a 10 (considerando 0 como muy en contra y 10 como muy a favor)^19^. Variables sociodemográficas como sexo y edad, variables relacionadas con la formación técnica o formación previa en PBE y el currículo cursado también fueron medidas.

Procedimientos: Para invitar a participar a los estudiantes, durante los periodos curriculares 1 y 2, el estudio se presentó de forma presencial por las investigadoras. Se expusieron los objetivos y procedimientos, así como la participación voluntaria. Para proteger de posible cohesión, se precisó que los resultados del estudio no influirían en la evaluación de ninguno de los cursos. En esa misma sesión y posterior a la firma del consentimiento informado, los estudiantes que cumplieron los criterios de selección fueron abordados para responder de forma auto diligenciada y por escrito el instrumento. Durante el periodo curricular 3, los estudiantes se abordaron a través del correo institucional o por grupos institucionales de difusión vía WhatsApp, para facilitar el acceso a la información debido a que no se desarrollaban clases presenciales durante la pandemia del COVID-19.

La información recopilada en físico fue digitada doblemente y tabulada por un asistente de investigación (persona con entrenamiento), mientras que la información del periodo curricular 3, fue registrada automáticamente en un formulario de Google. Finalmente, toda la información fue compilada en un archivo de Excel, el investigador realizó la limpieza y codificación para su análisis, y la versión final de la base de datos fue almacenada en el repositorio de acceso público de la UNAB^20^.

Análisis estadístico: Las características sociodemográficas, y la formación previa se describieron de acuerdo con el currículo cursado por los estudiantes. Las variables discretas se resumieron utilizando conteos y porcentajes, mientras que las variables continuas fueron resumidas con medianas y rango intercuartílico. La hipótesis de diferencias en la distribución de las variables discretas se evaluó con el estimador chi2 o prueba exacta de Fisher cuando las variables tenían conteos inferiores a 10 datos; y para evaluar la hipótesis de diferencias de medias en las variables continuas se realizó el análisis de varianza de una vía. Se determinó el puntaje global para cada dimensión del instrumento y para el total de la escala, presentando la mediana y rango intercuartílico para la puntuación. Estos puntajes se compararon de acuerdo con las características sociodemográficas, de formación previa y del currículo cursado. Todos los análisis fueron realizados en el software Stata v.12.0.

Aspectos éticos: Este estudio se clasifica como investigación sin riesgo según la resolución No 008430 de 1993 del Ministerio de Salud de Colombia. El estudio fue conducido de acuerdo con la declaración de Helsinki, por lo que solo fueron incluidos los estudiantes que aceptaron participar voluntariamente mediante la aceptación del consentimiento informado. La realización del estudio fue aprobada por el comité de ética institucional, actas No 064 de 2015 y No 135 de 2020.

## Resultados

Se incluyeron 125 estudiantes en este estudio; el 19,20% (n= 24) en el currículo SIN formación IE, el 44,00% (n= 55) en el currículo con formación IE, y el 36,80% (n= 46) en el currículo con formación en PBE.

Las características sociodemográficas y de formación previa de los estudiantes se presentan en la Tabla 1. En general, la mayoría de los estudiantes incluidos eran de género femenino (87,20%, n= 109); aunque la muestra del currículo con formación IE tenía una mayor proporción de mujeres en comparación a las demás. La edad mediana general fue de 22 años (Rango intercuartílico= 21-24 años), con una distribución similar en los tres periodos curriculares estudiados. La mayor frecuencia de estudiantes incluidos (65,60%, n= 85) cursaba el octavo semestre del programa, debido a que durante los tres periodos curriculares había un mayor número de estudiantes con matrícula activa para el octavo semestre (24 estudiantes para el currículo SIN formación IE, 34 estudiantes para el currículo con formación IE, 26 estudiantes para el currículo con formación en PBE) en comparación con el número de estudiantes con matrícula activa para el séptimo semestre (23 estudiantes para el currículo SIN formación IE, 21 estudiantes para el currículo con formación IE, 24 estudiantes para el currículo con formación en PBE). Adicionalmente, el total de la muestra recolectada para el periodo del currículo SIN formación en IE, corresponde a estudiantes que cursaban el octavo semestre debido a limitaciones para el acceso de las investigadoras a los estudiantes de séptimo semestre durante el periodo de medición. En general, más del 90% de la población elegible de los tres periodos curriculares fue incluida en este estudio.

El 39,84% (n= 49) de los estudiantes incluidos tenía alguna formación técnica previa (auxiliar de enfermería, laboratorio o salud ocupacional), siendo más prevalente en el grupo de estudiantes del currículo con formación IE. El 58,68% de los estudiantes manifestó recibir previo a la aplicación del instrumento, cursos, talleres, jornadas de actualización sobre PBE fuera de los cursos obligatorios del plan curricular; esta proporción fue estadísticamente superior para los estudiantes del currículo con formación en PBE.

**Tabla 1:** Características de los estudiantes incluidos de acuerdo con el currículo cursado

Características de los estudiantes	Total	Currículo SIN formación IE	Currículo formación IE	Currículo con formación en PBE	^p^
	125	24	55	46	
Género, n (%)					0.048*
Femenino	109 (87,20)	21 (87,50)	52 (94,55)	36 (78,26)	
Masculino	16 (12,80)	3 (12,50)	3 (5,45)	10 (21,74)	
Edad en años, mediana (RIC)	22 (21-24)	21 (20,50-23)	22 (21-26)	22 (21-23)	0,425^©^
Semestre, n (%)					<0,001*
7	43 (34,40)	-	21 (38,18)	22 (47,83)	
8	85 (65,60)	24 (100,00)	34 (61,82)	24 (52,17)	
Estudios técnicos previos, n (%)	49 (39,84)	11 (47,83)	29 (52,73)	9 (20,00)	0,002*
Formación previa en PBE, n (%)	71 (58,68)	13 (56,52)	25 (47,17)	33 (73,33)	0,031±

PBE= práctica basada en la evidencia, IE: Investigación en enfermería RIC: Rango Intercuartilico *Prueba exacta de Fisher, ± Prueba Chi2, ©Análisis de varianza de una vía

El puntaje global de la escala de competencias para la PBE fue alto para el total de estudiantes incluidos fue 4,08 (Rango intercuartílico= 3,84 -4,32) de un máximo de 5, con una distribución similar para las tres muestras en cada periodo curricular. Los menores puntajes se observaron en las dimensiones de conocimientos y habilidades de la escala, con medianas en los puntajes para el total de los estudiantes de 3,83 (Rango intercuartílico= 3,17- 4,17) y 3,67 (Rango intercuartílico= 3,33- 4,00), respectivamente. En la dimensión de habilidades estos puntajes no tuvieron diferencias estadísticamente significativas entre las muestras de los tres periodos curriculares, sin embargo, en la dimensión de conocimientos se observó un gradiente en el puntaje relacionado con las características del currículo cursado, siendo significativamente menor el puntaje para la muestra de estudiantes del currículo SIN formación IE (mediana= 3,00; rango intercuartílico= 3,00- 3,83). El puntaje obtenido en la dimensión de actitud hacia la PBE también tuvo diferencias estadísticamente significativas entre las muestras, observándose un mayor puntaje en la muestra de estudiantes con currículo con formación en IE (mediana= 4,69; rango intercuartílico= 4,23- 4,84). Ver Tabla 2.

**Tabla 2:** Distribución del puntaje obtenido por competencia de la PBE de acuerdo con el currículo cursado. Mediana (RIC)

Competencias	Total	Currículo SIN formación IE	Currículo formación IE	Currículo con formación en PBE	p©
	125	24	55	46	
Actitudes	4,38 (4,15- 4,69)	4,34 (4,19- 4,73)	4,69 (4,23- 4,84)	4,27 (4,07- 4,46)	0,002
Conocimientos	3,83 (3,17- 4,17)	3,00 (3,00- 3,83)	3,67 (3,33- 4,00)	4,00 (3,67- 4,33)	<0,001
Habilidades	3,67 (3,33- 4,00)	3,67 (3,16- 3,83)	3,67 (3,33- 4,00)	3,83 (3,50- 4,17)	0,117
Puntaje global	4,08 (3,84- 4,32)	4,00 (3,72- 4,12)	4,12 (3,84- 4,32)	4,08 (3,88- 4,36)	0,365

*PBE= práctica basada en la evidencia, IE: Investigación en enfermería. ©Análisis de varianza de una vía*

**Figura 1:**
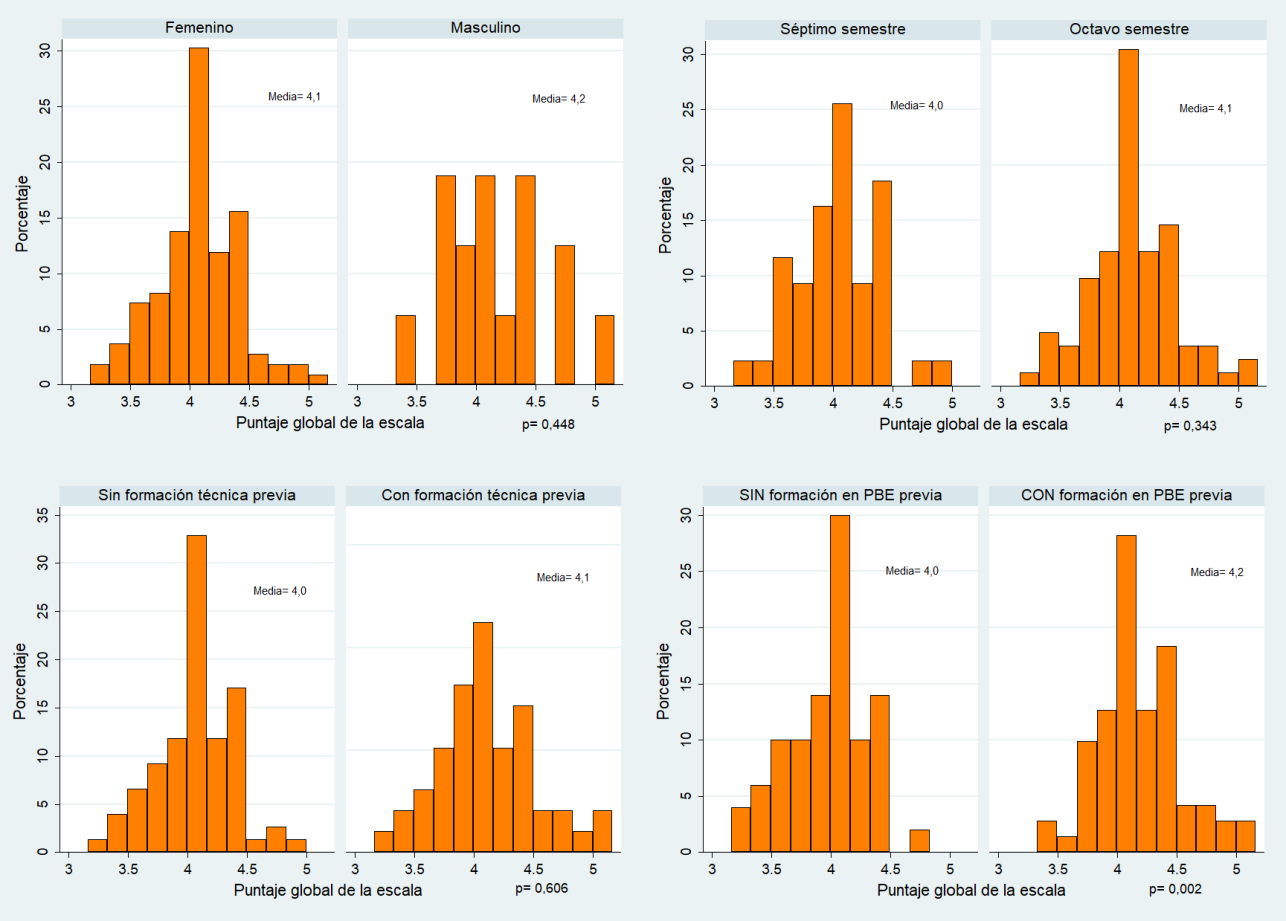
Distribución del puntaje global de la escala CACH-PBE de acuerdo con las características sociodemográficas y académicas de los estudiantes participantes

Las estudiantes mujeres tuvieron una media en el puntaje total de 4,07 (DE= 0,35), similar a la media del puntaje global obtenido por los estudiantes hombres (4,15 [DE= 0,44], valor p= 0,448). Estos puntajes también fueron muy similares al clasificar a los estudiantes de acuerdo con el semestre académico [media puntaje global séptimo semestre y octavo semestre de 4,04 (DE= 0,35) y 4,10 (DE= 0,37), respectivamente] y la formación técnica previa [media puntaje global con formación técnica previa de 4,10 (DE= 0,41) y sin formación técnica previa de 4,07(DE= 0,33)]. Sin embargo, esta distribución fue significativamente diferente en el grupo de estudiantes con formación complementaria previa en PBE versus quienes no tenían formación previa [media= 4,17 (DE= 0,35) versus media= 3,97 (DE= 0,35); valor p= 0,002,]. Ver Figura 1.

**Figura 2:**
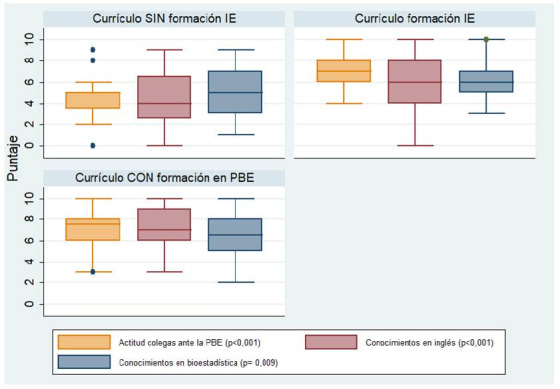
Percepción global de los estudiantes frente a las competencias para la PBE de acuerdo con el currículo cursado

En relación con la percepción global de los estudiantes frente a las competencias necesarias para la PBE, la Figura 2 presenta los puntajes con tendencia negativa que los estudiantes con currículo SIN formación IE tuvieron hacia aspectos como la actitud de los colegas hacia la PBE (mediana=5,00, rango intercuartílico= 3,50- 5,00), conocimientos en inglés (mediana= 4,00, rango intercuartílico 2,50 6,50) y conocimientos en bioestadística (mediana= 5,00, rango intercuartílico 3,00- 7,00). En estos aspectos se observó un cambio positivo en la percepción con un gradiente ascendente a lo largo de los periodos de ajustes curriculares. 

Otros ítems relacionados con la percepción frente a las competencias para la PBE tuvieron calificaciones positivas desde el primer periodo curricular, que se mantuvieron hasta el último. A excepción de la percepción frente al nivel de conocimientos en informática de oficina, se observó un aumento en los demás ítems evaluados, con un cambio significativo principalmente entre el currículo SIN formación IE y el currículo con formación en IE. (Ver Tabla 3)

**Tabla 3:** Distribución del puntaje obtenido para la percepción en los aspectos relacionados con la PBE de acuerdo con el currículo cursado. Mediana (RIC)

Percepciones	Total	Currículo SIN formación IE	Currículo formación IE	Currículo con formación en PBE	p©
	125	24	55	46	
Actitud ante la PBE	8,00 (7,00- 9,00)	6,50 (5,00- 8,00)	8,00 (7,00- 9,00)	9,00 (8,00- 9,00)	<0,001
Conocimientos teóricos sobre la PBE	7,00 (6,00- 8,00)	6,00 (4,50- 7,50)	7,00 (6,00- 8,00)	8,00 (7,00- 8,00)	<0,001
Habilidades prácticas para la aplicación de la PBE	7,00 (6,00- 8,00)	6,50 (5,00- 7,00)	7,00 (6,00- 8,00)	8,00 (7,00- 8,00)	0,011
Actitud ante la promoción actual de la PBE	8,00 (7,00- 9,00)	6,50 (5,00- 8,00)	9,00 (7,00- 9,00)	9,00 (7,00- 10,00)	<0,001
Nivel de conocimientos en informática	8,00 (7,00- 9,00)	8,00 (6,00- 8,00)	8,00 (7,00- 9,00)	8,00 (7,00- 9,00)	0,326

*PBE= práctica basada en la evidencia, IE: Investigación en enfermería ©Análisis de varianza de una vía*

## Discusión

Los ajustes curriculares realizados en la institución sugieren un mejoramiento de las competencias relacionadas con los conocimientos para la PBE en los estudiantes de enfermería, sin embargo, las competencias generales y en particular las habilidades para la PBE no mostraron diferencias entre los estudiantes observados en los tres periodos curriculares.

Algunos autores han descrito la relación entre el tiempo de las transformaciones curriculares y el desarrollo de competencias progresivas para la PBE, como un proceso escalonado cuyos resultados se reflejan al menos un ciclo curricular completo (4-5 años) después de su implementación^9^^,^^11^^,^^21^. En nuestra institución a partir del año 2014, se inició el proceso para obtener la certificación como organización académica comprometida con las mejores prácticas- BPSO otorgada por la Asociación de Enfermeras Registradas de Ontario (RNAO), y finalmente, en 2018 se obtuvo dicha certificación, lo que significó modificar procesos pedagógicos dentro de los micro currículos para incorporar las recomendaciones generadas por las guías de buenas prácticas clínicas de la asociación^22^, sin embargo, solo fue hasta el 2019, en el último ajuste curricular, en el cual se introdujeron adicional a los cursos de formación en investigación en enfermería, dos módulos de formación específica en PBE; es decir, que después de dos años de implementación de este último ajuste se generó la evaluación de la muestra de estudiantes incluidos en el último periodo de este estudio, lo cual podría explicar que la ausencia de diferencias significativas en el reporte de las competencias generales y habilidades para la PBE.

Adicionalmente, los ajustes en los contenidos curriculares no son el único elemento necesario para desarrollar competencias para la PBE, es indispensable presentar a los estudiantes de enfermería estrategias pedagógicas variadas y usarlas de forma simultánea con un enfoque multifacético que propicie principalmente los espacios de aprendizaje experiencial y que incluya el uso de tecnologías para la información y comunicación y la simulación^23^^-^^25^. Por otro lado, para dar una mayor sostenibilidad a la enseñanza de la PBE, los educadores requieren de capacitación y formación continua, y los profesionales de salud que laboran en las instituciones en donde los estudiantes desarrollan sus prácticas formativas deben tener prácticas basadas en la evidencia ^21^. En ese sentido, en el programa se está trabajando desde el año 2022, en un nuevo ajuste curricular que permita incorporar nuevas estrategias pedagógicas para transversalizar la enseñanza de la PBE, e impactar a estudiantes, educadores y profesionales desde el aula hasta los escenarios de práctica formativa.

En Latinoamérica, no existen otros estudios que describan el nivel de competencias para la PBE relacionado con diferentes contenidos curriculares en estudiantes de programas de enfermería. Los estudios realizados en dos instituciones de educación superior colombianas cuyos programas incluyen contenidos relacionados con investigación para enfermería, en los cuales usaron el instrumento CACH-PBE, identificaron un nivel general de competencias para la PBE inferior al encontrado en el presente estudio en la muestra de estudiantes con el currículo con formación en investigación en enfermería [En Cúcuta media para el puntaje global de 3,89 (Desviación estándar= 0,43), en Cali media= 3,45 (Desviación estándar= 0,40) y en nuestro estudio media= 4,11 (Desviación estándar= 0,39)]^17^^,^^18^. Estas diferencias a favor de los estudiantes del programa podrían explicarse por la adopción de las recomendaciones de las guías de buenas prácticas clínicas de la RNAO en los cursos específicos de la línea de enfermería tres años previos a la medición; aunque en su momento, la introducción de estas recomendaciones no incluyó dentro del currículo la formación epistemológica y teórica de preparación para la PBE, su uso en sí mismo representa una orientación genuina de este tipo de práctica. Así mismo, la estandarización de los contenidos, combinación de estrategias pedagógicas y un abordaje concertado entre los diferentes profesores de nuestro programa podrían relacionarse con este resultado. Adicionalmente, es importante considerar que una mayor proporción de los estudiantes de nuestro programa en comparación con la muestra de estudiantes evaluada en el Cúcuta (58,68% versus 41,90%)^18^, tuvo formación complementaria previa concerniente a PBE, particularmente los estudiantes del tercer periodo curricular, en donde de manera libre se ofertaron cursos, talleres o jornadas de capacitación sobre PBE como parte de las actividades de extensión como BPSO-RNAO de la institución.

En relación con la autopercepción de los estudiantes evaluados sobre aspectos relacionados con la PBE, los estudiantes con currículo SIN formación IE tuvieron una percepción con una tendencia negativa hacia aspectos como: actitud de los colegas hacia la PBE, conocimientos en inglés y conocimientos en bioestadística en comparación con los estudiantes del Currículo formación IE y Currículo con formación en PBE. Estos hallazgos no son inusuales dado que en el currículo inicial del programa no se incluían estos contenidos de forma específica, contando solo con dos cursos con elementos de investigación cuyo objetivo principal era introducir a los estudiantes a conceptos de la salud pública y condicionantes en salud. Aunque estos cursos incorporaron algunos contenidos relacionados con bioestadística, estos eran trabajados en una etapa muy inicial del programa de estudios (primer y segundo semestre) y no eran retomados posteriormente. Para el segundo semestre del 2019, el currículo del programa incluyó además de estos dos cursos iniciales, dos cursos de investigación específica en enfermería dirigidos a estudiantes de sexto y séptimo semestre (programa de formación de ocho semestres)^26^. Estos cursos adicionales permiten a los estudiantes tener una mayor exposición a los conceptos y oportunidades de práctica de los elementos de bioestadística a través del desarrollo de un proyecto de investigación y una menor disrupción dentro del currículo. Adicionalmente, para ese mismo periodo curricular los cursos de formación en inglés tuvieron algunas modificaciones en sus estrategias pedagógicas en comparación con el periodo curricular previo como una mayor interacción con docentes extranjeros, y la incorporación de prácticas en laboratorio de simulación para los cursos de inglés específico.

Este estudio tiene algunas fortalezas. En primer lugar, no existen otros estudios que identifiquen las competencias para la PBE en diferentes momentos de ajustes curriculares en programas de enfermería, por lo cual, el presente estudio representa una estrategia para evaluar el impacto de las reformas realizadas en el programa y podría resultar orientador para futuras modificaciones curriculares o la creación de nuevos programas de enfermería en otras instituciones. De otro lado, el instrumento CACH-PBE usado para evaluar las competencias para la PBE en los estudiantes de enfermería, ha sido validado y usado en otros contextos de Latinoamérica; estudios previos han mostrado que aunque es un instrumento diligenciado por los estudiantes a manera de auto reporte, su capacidad de discriminación es similar a la valoración objetiva que realizarían los educadores para determinar las competencias para la PBE^27^, por lo tanto, este instrumento podría aplicarse a futuro a manera de indicador de seguimiento. Este estudio también tiene algunas limitaciones; la muestra de estudiantes incluida no tuvo una estimación puntual y se restringió al número de estudiantes que se encontraban matriculados en el programa en el último año de estudios. Dado que se contaba en cada grupo de estudiantes evaluado con una medición inicial de su nivel de competencias para la PBE, las diferencias entre las características sociodemográficas de los estudiantes de cada muestra (incluyendo una mayor proporción de estudiantes con formación previa complementaria en PBE en el periodo del currículo con formación curricular en PBE), el carácter transversal de este estudio y otras variables no medidas en este estudio, no se puede asegurar que la adquisición de competencias esté directamente relacionada con las estrategias pedagógicas usadas en cada reforma curricular. Finalmente, al tratarse de una población específica, los resultados de este estudio podrían no ser aplicables a los estudiantes de los programas de formación en enfermería con currículos similares.

## Conclusión

Los ajustes curriculares realizados en nuestra institución sugieren un mejoramiento de las competencias relacionadas con los conocimientos para la práctica basada en la evidencia en estudiantes de enfermería, sin embargo, es necesario seguir trabajando en estrategias pedagógicas eficaces que permitan mejorar las competencias relacionadas con las habilidades para su implementación y adicionalmente las actitudes hacia la PBE. La información obtenida en este estudio provee orientación para futuras reformas curriculares, y permite realizar un seguimiento a los indicadores de formación en PBE establecidos por la institución. Las experiencias y los hallazgos descritos en este estudio también podrían guiar a los programas de enfermería de instituciones de educación superior en otros contextos para la incorporación de cursos o contenidos relacionados con la PBE dentro de su plan de estudios, recurriendo a diferentes estrategias pedagógicas en educación híbrida.
